# Partitioning of REE between calcite and carbonatitic melt containing P, S, Si at 650–900 °C and 100 MPa

**DOI:** 10.1038/s41598-022-07330-0

**Published:** 2022-02-28

**Authors:** Dmitry A. Chebotarev, Cora Wohlgemuth-Ueberwasser, Tong Hou

**Affiliations:** 1 V.S. Sobolev Institute of Geology and Mineralogy SB RAS, prosp. Akad. Koptyuga, 3, Novosibirsk, 630090 Russia; 2 State Key Laboratory of Geological Processes and Mineral Resources, China University of Geosciences, 29 Xueyuan Road, Beijing, 100083 China; 3 Helmholtz Centre Potsdam – German Research Centre for Geosciences GFZ, Telegrafenberg, 14473 Potsdam, Germany; 4 Institut Für Mineralogie, Leibniz Universtät Hannover, Callinstraße, 3, 30167 Hannover, Germany

**Keywords:** Geochemistry, Geology, Mineralogy, Petrology, Volcanology

## Abstract

Carbonatites host some unique ore deposits, especially REE, and fractional crystallization might be a potentially powerful mechanism for control enrichment of carbonatitic magmas by these metals to economically significant levels. At present, data on distribution coefficients of REE during fractional crystallization of carbonatitic melts at volcanic conditions are extremely scarce. Here we present an experimental study of REE partitioning between carbonatitic melts and calcite in the system CaCO_3_-Na_2_CO_3_ with varying amounts of P_2_O_5_, F, Cl, SiO_2_, SO_3_ at 650–900 °C and 100 MPa using cold-seal pressure vessels and LA-ICP-MS. The presence of phosphorus in the system generally increases the distribution coefficients but its effect decreases with increasing concentration. The temperature factor is high: at 770–900 °C D_REE_ ≥ 1, while at lower temperatures D_REE_ become below unity. Silicon also promotes the fractionation of REE into calcite, while sulfur contributes to retention of REE in the melt. Our results imply that calcite may impose significant control upon REE fractionation at the early stages of crystallization of carbonatitic magmas and might be a closest proxy for monitoring the REE content in initial melt.

## Introduction

Carbonatites are rare carbonate-dominated magmatic rocks that, in some cases, host economically significant deposits of REE and Nb, which are important for modern high-tech and “green” industry. More than 527 carbonatite occurrences have been recorded in the world so far^1^. However, only about 30 mineralized carbonatites host economic resources of REE^2,3^. The scientific and practical interest in carbonatites is great, but the processes of carbonatite formation and ore-grade enrichments of REE and Nb are far from being fully understood. Researchers identify the following mechanisms of carbonatite formation: (1) primary origin of carbonatitic magmas by partial melting of a carbonated mantle source^4–15^; (2) derivative origin from an homogeneous alkaline silicate magma by silicate-carbonate liquid immiscibility^16,17^; (3) derivative origin by extensive fractional crystallization of a carbonated silicate parental liquid^18,19^; (4) melting of oceanic sediments and material of the subducting plate, sublithospheric mantle and upper plate^20,21^.

Identification of exact processes responsible for the formation of carbonatitic magmas is hampered by widespread Na–K metasomatism (fenitization) associated with carbonatites, which manifests the loss of alkalis and volatiles from their parental magmas^22^. In addition, many (if not most) intrusive carbonatites are cumulates, dominated by calcite or dolomite, differing in composition from their parental magma^23^ and often affected by extensive textural and chemical re-equilibration after emplacement^24^.

As seen from experiments^14,25,26,27^, direct smelting from the mantle source and silicate-carbonate immiscibility are unable to provide concentration of REE to economically significant levels. Immiscibility experiments in various silicate–carbonate systems have demonstrated that Pb, Nb, Th, U and most of the REE preferentially partition into the silicate liquid, whereas Sr, Ba and F partition into the conjugate liquid carbonate phase^25,26,28^. This pattern of element partitioning is inconsistent with a high content of primary LREE- and HFSE-rich minerals in carbonatites: fluorapatite, fluorcarbonates, monazite, pyrochlore, etc. Thus, another evolutionary mechanism is needed for the enrichment of carbonatitic magmas in REE and HFSE, and fractional crystallization might be powerful driver for it.

The majority of experimental estimates of partition coefficients for REE have been obtained for main rock-forming minerals (olivine, pyroxene, garnet, amphibole and biotite), and some accessory minerals (such as rutile, apatite, perovskite, and baddeleyite) at P–T conditions of the upper mantle^27,29,30,31,32,33,34,35,36,37,38,39^. Experimental constraints on REE partitioning in carbonatitic magmas at crustal pressures are sparse. Because of low crystallization temperature and low viscosity of carbonatitic magmas, fractional crystallization can proceed in them up to shallow depths and control the distribution of REE and HFSE, leading to the formation of a deposit or a barren carbonatite body. Therefore, it is important to assess the effect of fractional crystallization at crustal conditions and to identify the influencing factors.

Carbonate minerals are the principal constituents of intrusive carbonatites: their content ranges from 50 modal %, which is accepted as a nominal threshold for this rock type^40^, to well over 90% in some varieties interpreted as cumulates^23^. Considering the high propensity of calcite to various postmagmatic changes, plasticity and recrystallization, observed trace element contents may be far from the original magmatic pattern. Hence, experimental modelling of trace element distribution between calcite and carbonatite melt at magmatic conditions is important.

Additional ligands such as F^−^, OH^−^, Cl^−^, SO_4_^2−^ and PO_4_^3−^ are likely to have strong effects upon the REE partitioning. In our previous work^41^ we carried out a pilot series of experiments to obtain distribution coefficients for REE and HFSE between calcite, fluorite and nominally dry carbonatitic melts in synthetic compositions with CaO at 37–54 wt.%, Na_2_O 7–24 wt.%, F 5–9 wt.%, P_2_O_5_ between 0 and 9.5 wt.% at 650–900 °C and 100 MPa. The distribution coefficients for individual REE in calcite in that study did not exceed 0.1 and in fluorite they were below 0.25. Total REE concentrations in calcites were at about 500–700 ppm. Such concentrations are normal for primary igneous calcites in carbonatites^42^. However, there are described primary igneous calcites with REE contents at 1400–2000 ppm (e.g., the Aley complex, Canada^42^). This study is aimed at a more detailed assessment of the effects of melt composition, and especially the concentrations of P_2_O_5_, SiO_2_ and SO_3_ components on the REE distribution between calcite and melt at 900–650 °C and 100 Mpa (Table 1).

**Table 1:** Nominal major and trace compositions of starting mixtures (in wt%).

Components (wt.%)	NCFP-7	NCFP-8	NCFP-9	NCP-1	NCP-2	NCPSi-1	NCPSi-2	NCPSi-3	NCPS-1	NCPS-2
SiO_2_	–	–	–	–	–	4.84	2.90	2.90	–	–
CaO	40.72	47.31	53.14	47.14	45.55	42.43	42.82	42.82	45.06	43.53
Na_2_O	18.57	11.37	5.78	20.31	21.64	21.68	22.38	21.89	21.34	22.61
F	10.12	9.19	10.62	0.03	0.01	0.02	0.01	0.01	0.02	0.01
Cl	–	–	–	0.02	0.01	0.02	0.01	0.01	0.02	0.01
SO_3_	–	–	–	–	–	–	–	–	2.50	2.50
P_2_O_5_	9.15	9.15	9.15	5.33	2.65	4.80	2.49	2.49	5.10	2.54
REE	0.40	0.40	0.40	0.50	0.05	0.45	0.05	0.47	0.48	0.05
HFSE	0.40	0.40	0.40	0.03	0.05	0.02	0.05	0.05	0.02	0.05
CO_2_	20.65	22.18	20.51	26.64	30.03	25.73	29.29	29.36	25.46	28.70
Total	100.00	100.00	100.00	100.00	100.00	100.00	100.00	100.00	100.00	100.00
(CaO + Na_2_O)/SiO_2_						13.24	22.45	22.28		
CaO/SiO						8.77	14.74	14.74		

## Results

**Figure 1:**
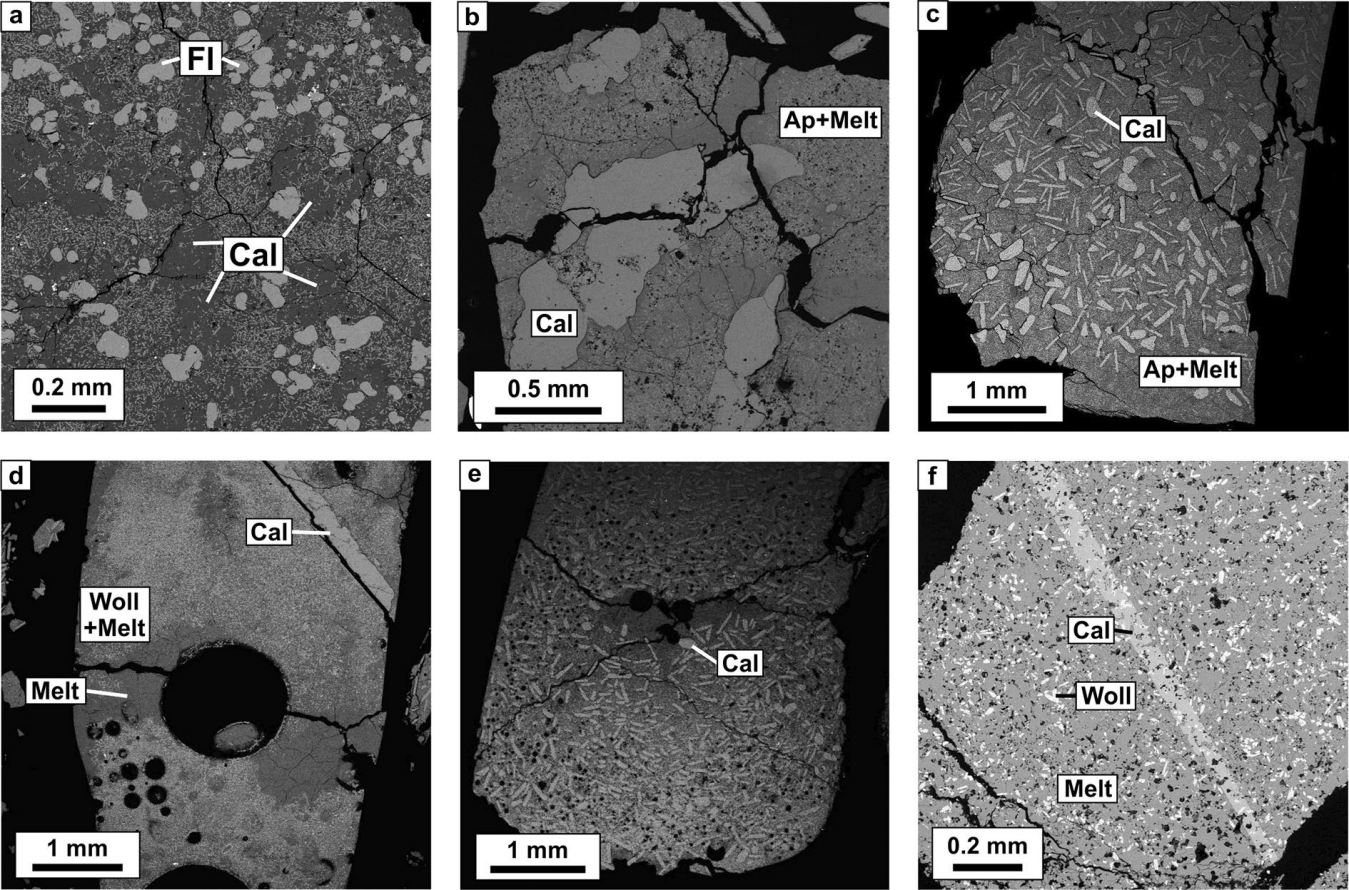
Morphology of calcites formed in experiments: (**a**) #3 NCFP-9; (**b**) #4 NCP-1; (**c**) #6 NCP-1; (**d**) #7 NCPSi-1; (**e**) #8 NCPS-1; (**f**) #10 NCPSi-3.

**Figure 2:**
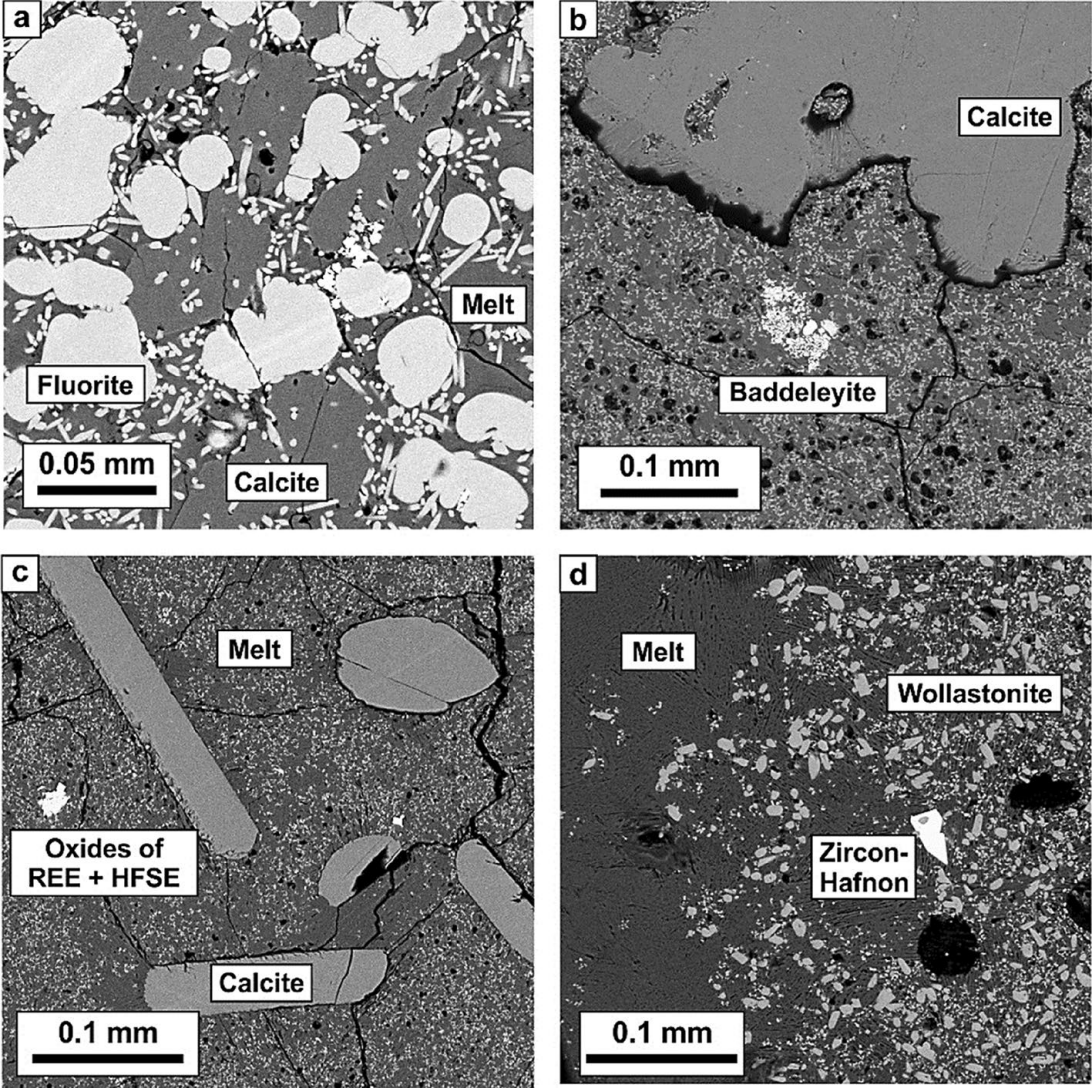
Textures of the experimental samples: (**a**) #3 NCFP-9; (**b**) #4 NCP-1; (**c**) #6 NCP-1; (**d**) #7 NCPSi-1.

Carbonatitic melts in all the experiments quenched as aggregates of fine-grained dendritic crystals with abundant fluid bubbles probably dominated by CO_2_ (Fig. 1, 2; Tables 2, 3). In phosphate-bearing samples, quenched melt is full of needle-shaped crystals of quench Ca-phosphate, presumably apatite (Figs. 1a, Fig. 2a–c). In experiments with SiO_2_ fine crystals of wollastonite and Zr-Hf-silicate (zircon-hafnon) are also formed (Fig. 2d; Table 2). Oxides of HFSE and REE in all samples form aggregates of fine crystals with complex composition (baddeleyite, perovskite-lueshite solid solutions and crystals of pyrochlore supergroup) (Fig. 2a–c). The crystals are few microns in size and are not suitable for representative analyzes by microprobe or LA-ICP-MS. Rounded, irregularly shaped, drop-like fluorite crystals are formed in samples with high content of F (Fig. 2a; Table 2).

**Table 2:** Experimental conditions and run products.

Run #	Mixture	Temperature (°C)	Duration (h)	Phases
1	NCFP-7	760	26	Melt, fluid, calcite
2	NCFP-8	900–850	20	Melt, fluid, calcite
3	NCFP-9	900–650	141	Melt, fluid, calcite, fluorite, baddeleyite, pyrochlore
4	NCP-1	900–830	44	Melt, calcite, baddeleyite
5	NCPS-1	900–830	25	Melt, fluid, calcite
6	NCP-1	820–770	55	Melt, fluid, calcite, REE and HFSE oxides
7	NCPSi-1	820–770	55	Melt, fluid, calcite, wollastonite, zircon-hafnon
8	NCPS-1	820–770	55	Melt, fluid, calcite, baddeleyite, pyrochlore
9	NCP-2	820–770	64	Melt, fluid, calcite, Hf-oxide, REE and HFSE oxide
10	NCPSi-2	820–770	64	Melt, fluid, calcite, wollastonite, zircon-hafnon
11	NCPS-2	820–770	64	Melt, fluid, calcite, REE and HFSE oxides
12	NCPSi-3	900–760	52	Melt, fluid, calcite, wollastonite

**Table 3:** Major composition of run products (in wt.%).

Run #	Mixture	Phase	Na_2_O	CaO	SiO_2_	P_2_O_5_	SO_3_	F	Cl	(Cl + F_2_) = –O	Total
1	NCFP-1	Melt (3)	12 (2)	48 (2)	–	12 (3)	–	9.7 (9)	–	4.10	77.00
		Calcite (5)	0.06 (3)	57 (1)	–	–	–	–	–	–	57.06
2	NCFP-3	Melt (6)	6 (1)	54 (1)	–	11 (2)	–	11 (1)	–	4.50	77.00
		Calcite (4)	0.09 (3)	56.1 (0)	–	–	–	–	–	–	56.20
3	NCFP-4	Melt (19)	20 (2)	35.9 (4)	–	0.6 (1)	–	10.4 (2)	–	–	67.00
		Calcite (16)	–	54.5 (3)	–	–	–	–	–	–	54.60
4	NCP-1	Fluorite (17)	–	71.6 (4)	–	1 (1)	–	48.4 (7)	–	20.40	100.00
		Melt (9)	20.5 (17)	35.9 (4)	–	0.6 (1)	–	10.4 (2)	–	–	67.40
		Calcite (10)	–	53.4 (6)	–	–	–	–	–	–	53.40
5	NCPS-1	Melt (12)	20 (2)	35 (2)	–	6 (4)	0.8 (3)	0.63 (9)	0.3 (2)	0.40	62.86
		Calcite (7)	–	54 (0)	–	–	–	–	–	–	54.00
6	NCP-1	Melt (9)	22.2 (7)	32 (1)	–	6 (1)	–	0.66 (7)	0.42 (8)	0.47	60.81
		Calcite (11)	–	54 (0)	–	–	–	–	–	–	54.00
7	NCPSi-1	Melt (2)	23 (2)	31 (2)	2 (3)	2 (2)	–	0.6 (4)	0.4 (1)	0.43	58.82
		Calcite (7)	–	54 (0)	–	–	–	–	–	–	54.00
8	NCPS-1	Melt (17)	23 (2)	31 (2)	–	4 (4)	1 (0)	0.66 (8)	0.17 (2)	0.36	59.92
		Calcite (8)	–	53.3 (5)	–	–	–	–	–	–	53.30
9	NCP-3	Melt (10)	25.6 (9)	30.6 (9)	–	3 (2)	–	–	0.18 (1)	0.08	58.92
		Calcite (8)	0.4 (3)	54.7 (2)	–	–	–	–	–	–	55.10
10	NCPSi-3	Melt (11)	21 (3)	30.7 (7)	1.9 (6)	–	–	–	0.03 (4)	0.01	53.99
		Calcite (4)	–	54.6 (3)	–	–	–	–	–	–	54.60
11	NCPS-3	Melt (16)	25 (2)	31 (2)	–	3 (4)	0.7 (1)	0.45 (1)	0.07 (2)	0.22	58.97
		Calcite (9)	0.27 (6)	54.6 (7)	–	–	–	–	–	–	54.80
12	NCPSi-4	Melt (8)	26 (2)	29.8 (4)	–	2 (3)	–	–	0.06 (0)	0.03	56.97
		Calcite (6)	–	53.7 (4)	–	–	–	–	–	–	53.70

The numbers in the parentheses next to the phase name indicates the number of analyzes. The numbers in the parentheses next to the analyzes are the standard deviation and reported as the least unit cited. For example, 55.50 (64) should be read as 55.50 ± 0.64 wt.%.

Calcite has various morphology: rhombohedral or prismatic (Fig. 1a, 2a), amorphous (Fig. 1b), needle-like (Fig. 1d,f) and tabular rounded crystals (laths) up to 250 µm in most experiments (Figs. 1c,e, 2c), which are typical for early generations of calcite in carbonatites^43^. The amount of calcite crystals in some samples is so high that they form a crystal mush at the bottom of the samples (Fig. 1c,e). Calcite major element composition is close to stoichiometric (Table 3).

**Figure 3:**
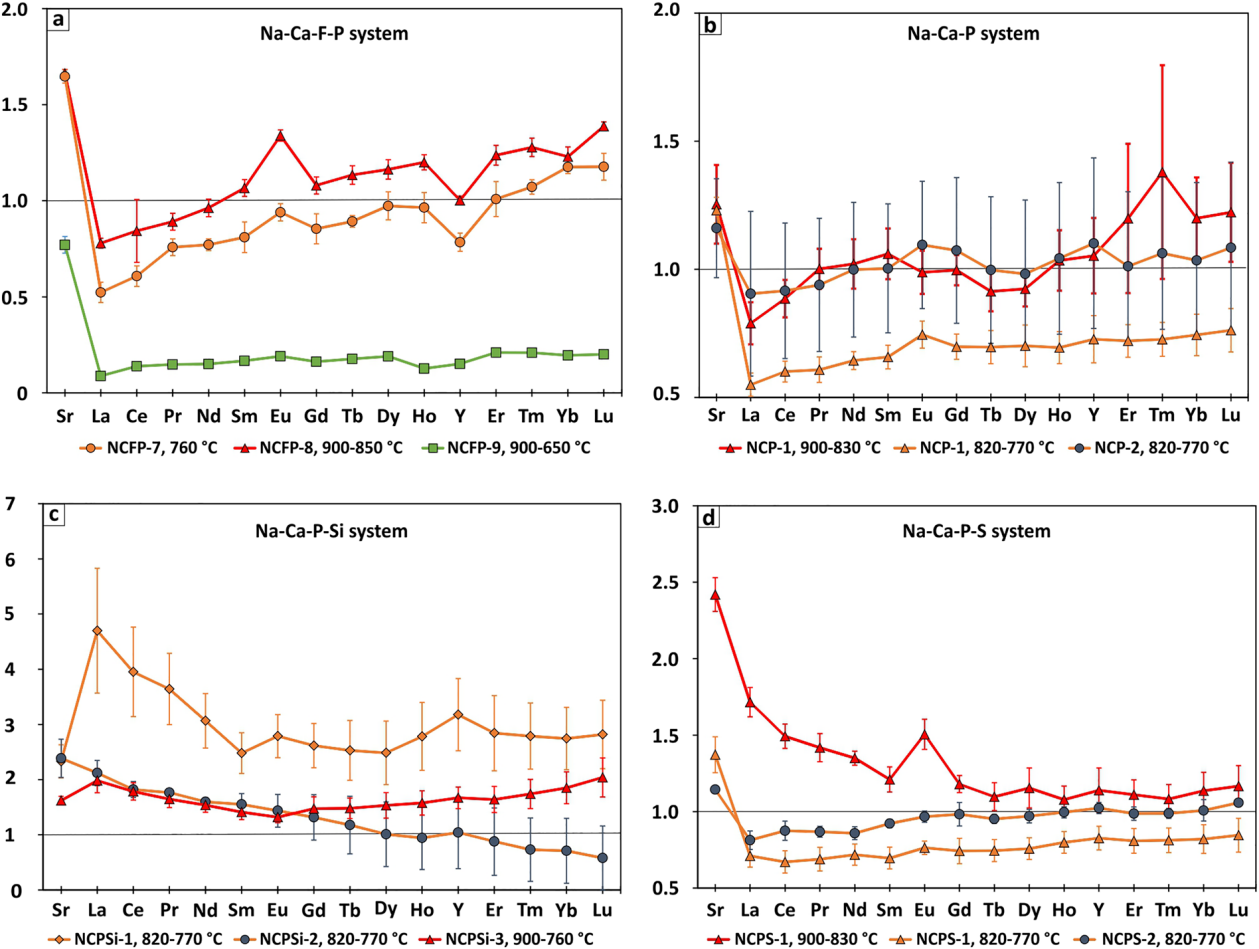
The average partition coefficients of REE with the standard deviation as error bars for calcites.

Trace element composition of the run products presented in Table 4. Partition coefficients were calculated according to the Nernst formula as mass ratios of element content in a crystalline phase to its content in the melt: D_Element_ = C^Crystal^/C^Melt^ (Table 5). D_Sr_ is > 1 for all samples (Fig. 3). In all experiments D_Sr_ >> D_REE_, except NCPSi mixtures. Almost all D_REE_ plots show a positive Eu and Y anomalies. In general, the slope of the D_REE_ plots is positive, with the exception of NCPSi samples.

**Table 4:** Trace element composition of run products (in ppm).

Run #	Mixture	Phase	Sr	La	Ce	Pr	Nd	Sm	Eu	Gd	Tb	Dy	Ho	Y	Er	Tm	Yb	Lu
1	NCFP-1	Melt (5)	450 (24)	183 (10)	155 (9)	160 (10)	153 (8)	178 (7)	155 (8)	161 (10)	155 (7)	165 (11)	173 (8)	156 (8)	172 (7)	172 (7)	177 (10)	163 (11)
		Calcite (5)	741 (24)	95 (10)	94 (9)	121 (10)	118 (8)	144 (7)	145 (8)	138 (10)	138 (7)	160 (11)	166 (8)	122 (8)	174 (7)	184 (7)	208 (10)	192 (11)
2	NCFP-3	Melt (5)	426 (9)	132 (4)	120 (6)	137 (10)	126 (6)	134 (7)	135 (4)	132 (7)	126 (5)	135 (8)	135 (5)	129 (2)	136 (7)	138 (3)	175 (12)	128 (3)
		Calcite (3)	708 (6)	103 (5)	101 (6)	122 (8)	121 (9)	143 (7)	181 (6)	142 (7)	143 (9)	157 (11)	161 (8)	129 (2)	168 (10)	176 (12)	215 (10)	178 (2)
3	NCFP-4	Melt (5)	703 (60)	618 (51)	389 (13)	427 (13)	444 (29)	435 (34)	499 (40)	453 (34)	438 (27)	438 (20)	665 (62)	474 (43)	443 (11)	471 (14)	535 (24)	552 (34)
		Calcite (5)	520 (73)	85 (44)	79 (38)	90 (42)	95 (44)	95 (37)	123 (43)	96 (37)	97 (32)	102 (30)	110 (29)	83 (20)	110 (28)	115 (27)	119 (25)	123 (22)
		Fluorite (3)	195 (47)	bdl	bdl	11 (11)	9 (3)	29 (11)	33 (1)	55 (25)	51 (22)	56 (22)	109 (79)	152 (94)	46 (26)	85 (65)	48 (13)	55 (24)
		Ca-phosphate (1)	581	116	103	114	116	118	154	129	128	132	141	85	134	136	139	134
4	NCP-1	Melt (9)	126 (18)	274 (29)	225 (29)	261 (54)	285 (59)	282 (52)	289 (48)	268 (47)	258 (51)	247 (51)	253 (35)	268 (41)	260 (11)	286 (47)	308 (63)	314 (62)
		Calcite (5)	150 (28)	248 (40)	196 (22)	232 (10)	265 (25)	279 (34)	292 (56)	296 (57)	277 (52)	270 (48)	292 (52)	281 (32)	303 (64)	336 (84)	318 (32)	343 (31)
5	NCPS-1	Melt (5)	80 (3)	169 (6)	161 (8)	179 (8)	191 (6)	204 (10)	197 (9)	201 (9)	196 (8)	193 (9)	188 (9)	176 (10)	183 (9)	182 (11)	186 (14)	182 (12)
		Calcite (5)	180 (21)	262 (42)	214 (39)	233 (30)	238 (27)	226 (26)	264 (38)	216 (29)	203 (21)	207 (28)	193 (19)	190 (22)	189 (24)	185 (20)	193 (30)	200 (27)
6	NCP-1	Melt (5)	109 (4)	268 (19)	219 (14)	244 (16)	257 (14)	262 (13)	263 (14)	265 (18)	260 (15)	262 (14)	262 (15)	253 (15)	252 (13)	256 (16)	275 (21)	277 (18)
		Calcite (5)	137 (6)	147 (28)	132 (28)	148 (32)	166 (33)	172 (42)	196 (36)	185 (46)	181 (49)	184 (51)	182 (48)	184 (45)	182 (50)	186 (54)	205 (70)	211 (62)
7	NCPSi-1	Melt (3)	124 (15)	210 (47)	167 (24)	202 (28)	221 (17)	240 (56)	234 (28)	243 (26)	224 (32)	212 (27)	225 (40)	222 (38)	226 (37)	235 (45)	266 (62)	272 (62)
		Calcite (3)	156 (4)	125 (2)	84 (1)	86 (1)	82 (1)	71 (1)	78 (2)	70 (2)	65 (0)	66 (1)	69 (2)	72 (1)	65 (0)	64 (1)	65 (1)	67 (1)
8	NCPS-1	Melt (5)	87 (2)	204 (11)	178 (10)	199 (9)	208 (7)	220 (10)	223 (9)	222 (8)	218 (10)	215 (10)	219 (11)	201 (12)	208 (13)	206 (17)	214 (21)	219 (26)
		Calcite (5)	119 (8)	147 (14)	122 (12)	139 (12)	153 (15)	155 (15)	173 (13)	167 (17)	163 (17)	164 (16)	176 (17)	166 (15)	167 (17)	167 (15)	175 (17)	183 (18)
9	NCP-3	Melt (5)	67 (8)	27 (6)	21 (4)	24 (4)	27 (4)	29 (4)	28 (4)	27 (4)	26 (6)	26 (6)	25 (5)	23 (5)	23 (5)	23 (5)	24 (5)	24 (5)
		Calcite (5)	78 (8)	24 (6)	20 (4)	22 (5)	27 (6)	29 (6)	31 (6)	29 (6)	26 (5)	26 (5)	26 (5)	25 (6)	23 (4)	24 (4)	25 (5)	26 (6)
10	NCPSi-3	Melt (5)	57 (6)	17 (2)	14 (1)	16 (0)	17 (0)	17 (2)	21 (4)	21 (7)	21 (9)	20 (12)	21 (13)	24 (15)	20 (14)	21 (16)	21 (17)	26 (26)
		Calcite (4)	136 (14)	35 (2)	26 (0)	28 (0)	27 (1)	27 (1)	31 (1)	28 (0)	25 (1)	21 (0)	20 (1)	25 (2)	17 (1)	15 (1)	15 (2)	15 (2)
11	NCPS-3	Melt (5)	69 (1)	27 (2)	20 (1)	24 (1)	27 (1)	27 (1)	27 (1)	26 (1)	27 (1)	26 (1)	27 (1)	25 (1)	25 (1)	24 (1)	26 (1)	26 (0)
		Calcite (5)	89 (18)	29 (12)	23 (9)	27 (12)	31 (14)	33 (14)	33 (11)	34 (15)	32 (12)	32 (11)	33 (11)	30 (8)	31 (10)	28 (8)	32 (10)	33 (8)
12	NCPSi-4	Melt (5)	46 (2)	107 (8)	92 (6)	106 (8)	116 (8)	117 (13)	136 (10)	112 (18)	105 (19)	96 (19)	95 (24)	87 (22)	83 (26)	81 (32)	77 (32)	73 (37)
		Calcite (5)	73 (5)	200 (17)	154 (12)	163 (12)	169 (10)	152 (7)	168 (12)	150 (10)	138 (6)	132 (6)	129 (5)	126 (2)	114 (5)	111 (5)	110 (5)	108 (6)

The numbers in the parentheses next to the phase name indicates the number of analyzes. The numbers in the parentheses next to the analyzes are the standard deviation. For example, 182 (48) should be read as 182 ± 48 ppm.

**Table 5:** Calcite-melt partition coefficients of trace elements.

Element	1		2		3		4		5		6		7		8		9		10		11		12	
	NCFP-7		NCFP-8		NCFP-9		NCP-1		NCPS-1		NCP-1		NCPSi-1		NCPS-1		NCP-2		NCPSi-2		NCPS-2		NCPSi-3	
	D_REE_	σ	D_REE_	σ	D_REE_	σ	D_REE_	σ	D_REE_	σ	D_REE_	σ	D_REE_	σ	D_REE_	σ	D_REE_	σ	D_REE_	σ	D_REE_	σ	D_REE_	σ
Sr	1.65	0.07	1.66	0.04	0.77	0.04	1.25	0.15	2.42	0.11	1.23	0.05	2.33	0.30	1.37	0.12	1.16	0.19	2.39	0.35	1.14	0.01	1.62	0.08
La	0.52	0.11	0.78	0.04	0.09	0.01	0.79	0.08	1.72	0.10	0.55	0.05	4.70	1.13	0.71	0.07	0.90	0.32	2.12	0.23	0.81	0.06	1.98	0.22
Ce	0.61	0.11	0.84	0.06	0.14	0.01	0.89	0.07	1.49	0.08	0.60	0.04	3.95	0.81	0.67	0.07	0.92	0.26	1.82	0.14	0.87	0.06	1.78	0.16
Pr	0.76	0.09	0.89	0.09	0.15	0.01	1.00	0.08	1.42	0.09	0.61	0.05	3.64	0.65	0.69	0.08	0.94	0.26	1.76	0.04	0.87	0.04	1.65	0.16
Nd	0.77	0.06	0.96	0.08	0.15	0.02	1.02	0.10	1.35	0.05	0.64	0.03	3.07	0.49	0.72	0.07	1.00	0.26	1.60	0.08	0.86	0.04	1.54	0.13
Sm	0.81	0.16	1.07	0.08	0.17	0.02	1.06	0.10	1.21	0.08	0.66	0.05	2.48	0.37	0.70	0.07	1.00	0.25	1.55	0.19	0.92	0.03	1.41	0.13
Eu	0.94	0.09	1.34	0.06	0.19	0.02	0.99	0.08	1.50	0.10	0.75	0.05	2.79	0.39	0.76	0.04	1.10	0.25	1.44	0.30	0.97	0.04	1.32	0.10
Gd	0.85	0.16	1.08	0.08	0.16	0.02	1.00	0.06	1.18	0.06	0.70	0.05	2.61	0.40	0.74	0.08	1.07	0.28	1.32	0.41	0.98	0.08	1.47	0.22
Tb	0.89	0.06	1.13	0.08	0.18	0.01	0.91	0.08	1.10	0.09	0.70	0.06	2.53	0.54	0.74	0.07	1.00	0.29	1.18	0.52	0.95	0.03	1.48	0.19
Dy	0.97	0.15	1.16	0.11	0.19	0.01	0.92	0.07	1.15	0.13	0.70	0.08	2.48	0.57	0.76	0.07	0.98	0.29	1.01	0.58	0.97	0.04	1.53	0.23
Ho	0.96	0.15	1.20	0.07	0.13	0.01	1.03	0.12	1.08	0.09	0.69	0.06	2.78	0.61	0.80	0.07	1.04	0.30	0.94	0.57	1.00	0.04	1.58	0.22
Y	0.78	0.09	1.00	0.03	0.15	0.02	1.05	0.15	1.14	0.15	0.73	0.09	3.18	0.66	0.83	0.08	1.10	0.33	1.04	0.65	1.02	0.04	1.67	0.20
Er	1.01	0.18	1.24	0.10	0.21	0.01	1.20	0.29	1.11	0.10	0.72	0.06	2.84	0.68	0.81	0.08	1.01	0.29	0.88	0.61	0.99	0.04	1.64	0.24
Tm	1.07	0.07	1.28	0.09	0.21	0.02	1.38	0.42	1.08	0.10	0.73	0.07	2.79	0.60	0.81	0.08	1.06	0.30	0.73	0.57	0.99	0.03	1.74	0.26
Yb	1.18	0.07	1.23	0.10	0.20	0.02	1.20	0.16	1.14	0.12	0.74	0.08	2.74	0.56	0.82	0.09	1.04	0.30	0.71	0.59	1.01	0.07	1.85	0.29
Lu	1.18	0.14	1.39	0.04	0.20	0.02	1.22	0.19	1.17	0.13	0.76	0.08	2.82	0.62	0.85	0.11	1.08	0.33	0.58	0.58	1.06	0.02	2.04	0.35

σ the standard deviation.

D_REE_ of calcites, published in our previous work^41^, are close to zero: 0.02–0.04 in NCF samples and 0.04–0.1 in sample with NCFP-6. In experiments with the NCFP mixtures, presented in this work, the averages of D_REE_ are much higher: 0.1–0.2 and 0.52–1.39 (Fig. 3a). The averages of D_REE_ in NCP samples vary in 0.55–1.4, but taking into account the analytical uncertainty for each individual element, it is almost the same (Fig. 3b). In experiments at highest temperatures the highest D_REE_ are observed for both mixtures.

NCPSi samples demonstrate highest D_REE_ with large variations (Fig. 3c). #7 NCPSi-1 sample demonstrates the highest DREE among them, while D_REE_ of #10 NCPSi-2 and #12 NCPSi-3 is almost equal. This is probably directly related to the content of SiO_2_ and P_2_O_5_ in the samples.

The graphs of D_REE_ of NCPS samples are almost flat, with a slight positive slope. The average D_LREE_ in #5 NCPS-1 vary in 1.2–1.7 with strong Eu anomaly, while the coefficients of other elements are about 1.2 and almost equal with #11 NCPS-2.

## Discussion

### Factors influencing the partitioning of REE into calcite in carbonatite systems

As seen from natural carbonatite samples, the early generations of calcites are characterized by increased content of cations, which size doesn’t allow stoichiometrically replace Ca^2+^ at crustal conditions: Mg, Sr, Ba are most abundant among them^43,44^. Sometimes it leads to formation of Ca-Ba-Sr “protocarbonates”, which are unstable and decay into calcite and baritocalcite in the form of a solid solution. Consequently, elevated temperatures favor the entry of cations, whose size precludes their unlimited substitution for Ca^2+^. Along with these cations, REE may also enter the structure of calcite, which is also noted in natural samples: for example, the positive correlation between Ba, Pb, and LREE in early calcites of the Aley complex (Canada)^42,43^. The strong effect of temperature is confirmed by our experiments: in most experiments D_REE_ > 1 at high temperatures (900–820 °C), while it is about unity or less at lower temperatures^41^.

As follows from a comparison with our previous experiments^41^, the presence of small amounts of phosphorus may be another positive and key factor for the fractionation of REE into calcite in carbonatite systems with high Na_2_O content. The presence SiO_2_ in small amounts in the system may be another powerful driver for incorporating D_REE_ into calcite. Sulfur, on the contrary, may promotes the dissolution (retention) of REE in the melt. However, to evaluate the total effect of these and other parameters (such as F, Cl) additional experiments are required.

### Calcite as a monitor of REE

Our data show that, under the combination of some factors at the magmatic stage of evolution of carbonatite magma, calcite can retain a significant gross budget of REE and may be a closest proxy for estimation of initial REE content in the melt. For example, at high temperatures, in local areas of a carbonatite body, or when there is a lack of material for fractionation of early traditional mineral concentrators of a large amount of REE (such as apatite, monazite, etc.) even at low concentration of REE in the melt.

High susceptibility of calcite to postmagmatic changes, plasticity and recrystallization creates the possibility of releasing from calcite large bulk amounts of REE, Sr and Ba and their subsequent redeposition as a result of interaction with later melts or solutions or as a result of metamorphism. According to various estimates^42,43^, such processes can occur at shallow depths already at temperatures of 400–600 and even 730 °C, which can lead to active loss of REE by calcite, overprinting his textures, and transfer and redeposition of REE far from it due to weakening of the carbonatite body by porous fluids. These events can also be aggravated by recrystallization of calcite during plastic deformation under a stress conditions.

Patterns of such phenomena occur, for example, in calcites from Afrikanda and Murun carbonatites (Russia) and Bearpaw Mts (USA)^43^: diffusion-induced zoning in primary calcite involving a decrease in Mn, Sr , Ba and REE along grain boundaries and fractures; the Sr and REE released from calcite are precipitated interstitially as strontianite, baritocalcite, Ba-Sr-Ca-carbonates, barite, burbankite and ancylite.

## Methods

Experimental mixtures were composed of reagent-grade CaCO_3_, Na_2_CO_3_, Ca_3_(PO_4_)_2_, CaF_2_, Na_2_Si_2_O_5_ and Na_2_SO_4_ and doped with mixtures of trace elements: Zr, Hf, Nb, Ta, Ti, Sr, W, Mo (HFSE mixture) and all REE, including Sr and Y (REE mixture) (Table 1). Both mixtures of trace elements were composed of equal weight proportions of reagent-grade CeF_2_, SrCO_3_ and pure oxides of other individual elements. Pure reagents were dried at 180 °C overnight, mixed under acetone in agate mortar and dried again. The mixtures of NCFP type are direct analogues of our mixtures, used in previous work^41^. About 45–55 mg starting mixtures were put in gold capsules and sealed by arc-welding in the flow of Ar.

Experiments were performed at pressure of 100 MPa and temperature range of 650–900 °C in rapid-quench cold-seal pressure vessels at the German Research Centre for Geosciences (GFZ Potsdam). The temperature range is close to the parameters for nature cooling systems of carbonatitic melts. A detailed description of this type of the vessels described in^45^. The autoclaves at GFZ Potsdam are made of the Ni–Cr alloy Vakumelt ATS 290-G (ThyssenKrupp AG). Oxygen fugacity was not controlled, but believed to have been close to that of the Ni–NiO equilibrium, buffered by oxidation reactions of water, used as pressure medium, and the Ni–Cr alloy of the autoclave. Temperature measured by external Ni–CrNi thermocouple, calibrated against the melting temperature of gold. Temperature measurements are corrected for a temperature gradient inside the autoclaves, which was measured using a second inner thermocouple during the initial calibration of the vessels. The external thermocouple has an uncertainty of approximately ± 1 °C, and the total uncertainty of temperature measurements, including the uncertainties due to the temperature gradients, is estimated to be ± 5 °C. Pressure is measured by transducers, and results were checked against a pressure gauge. The transducers and gauge are factory calibrated and have an accuracy of better than ± 0.1 MPa. Run times varied from 20 to 141 h. In experiments with decreasing temperature, the temperature lowered gradually, with decrease of 5–7 °C in every 1–1.5 h. Samples were kept for at least 10 h at the final temperature before quenching. The quenching of the experimental samples in such apparatus is isobaric and less than a second.

After the experiments, samples were mounted in epoxy resin and polished with diamond polishing pastes without water to avoid dissolution of alkalis. Major components of the run products were analyzed using Cameca SX-50 and SX-100 electron microprobes in the GFZ Potsdam and energy-dispersive spectrometry (EDS) in combination with back-scattered electron imaging (BSE) using a MIRA 3 LMU SEM (TESCAN Ltd.) equipped with an INCA Energy 450 XMax 80 microanalysis system (Oxford Instruments Ltd.) in the Analytical Center for multi-elemental and isotope research SB RAS (Novosibirsk, Russia).

Microprobe analyses were performed in WDS mode with a 10 nA beam current and accelerating voltage of 15 kV. Counting time for F (analyzed with TAP crystal) was 40 s. For all other elements it was set to 20 s on peak and 10 s on background. These parameters are benign to avoid any damage to carbonates or melt. The following synthetic and natural standards were used for the calibration: wollastonite (Ca), albite and jadeite (Na), apatite (P), LiF (F). Quenched melts were analyzed with a defocused beam (beam diameter of 20–40 µm). At least five point analyses were performed on each phase to obtain statistically representative averages.

For EDS analyses the samples were coated with a 25 µm conductive carbon coating. EDS analyses were made at low vacuum of 60–80 Pa, an accelerating voltage of 20 kV, a probe current of 1 nA, and accumulation time of 20 s. The simple compounds and metals were used as reference samples for most of the elements: SiO_2_ (Si-Kα and O-Kα), diopside (Ca-Kα), albite (Na-Kα), Ca_2_P_2_O_7_ (P-Kα), BaF_2_ (F-Kα), pyrite (S-Kα), SrF_2_ (Sr-Kα) and various (LREE)PO_4_ (LREE-Lα). Correction for matrix effects was made by the XPP algorithm, implemented in the software of the microanalysis system. Metallic Co served for quantitative optimization (normalization to probe current and energy calibration of the spectrometer).

Trace elements were measured by LA-ICP-QQQ-MS applying a Geolas Compex Pro 193 nm excimer laser coupled to a Thermo iCAP TQ mass spectrometer in the GFZ Potsdam. Analytical conditions comprised a spot size of 24 µm, repetition rate of 8 Hz, and laser energy density of 5 J cm^−2^. NIST SRM 610 was used as external standard and Ca as derived from EPMA as internal standard. BCR-2G was analyzed as the validation material known-unknown for accuracy control and its trace element concentrations are in agreement with published reference datavalues at < 10% 2σ RSD. Each analysis comprised 20 s background, 40 s ablation and 20 s wash-out. Data was processed using the trace elements IS data reduction scheme^46^ in iolite 3.63^47^.

Experimental experience gained with carbonatite systems under these conditions by us and by other researchers shows that the chosen duration of experiments is sufficient to achieve equilibrium in experiments with synthetic carbonatites due to the high rate of kinetic processes in them, especially with small experimental samples. Moreover, most of the duration of the experiments (from tens of hours to days) took place at the final temperature of the experiments to achieve equilibrium before quenching. During the subsequent analysis of the samples both by scanning microscopy and LA-ICP-MS, we did not find any zoning in the content of either the main components or rare elements in the center and on the periphery of the crystal phases.
